# A novel particle filtering method for estimation of pulse pressure variation during spontaneous breathing

**DOI:** 10.1186/s12938-016-0214-x

**Published:** 2016-08-11

**Authors:** Sunghan Kim, Fouzia Noor, Mateo Aboy, James McNames

**Affiliations:** 1 Biomedical Instrumentation & Data Analysis Laboratory, East Carolina University, Greenville, NC USA; 2 Electrical Engineering Department, Oregon Institute of Technology, Portland, OR USA; 3 Biomedical Signal Processing Laboratory, Portland State University, Portland, OR USA

**Keywords:** Extended Kalman filter, *a-posteriori* distribution, Maximum *a-posteriori* estimation, Marginalized particle filter, Multi-harmonic signal

## Abstract

**Background:**

We describe the first automatic algorithm designed to estimate the pulse pressure variation ($\text {PPV}$) from arterial blood pressure (ABP) signals under spontaneous breathing conditions. While currently there are a few publicly available algorithms to automatically estimate $\text {PPV}$ accurately and reliably in mechanically ventilated subjects, at the moment there is no automatic algorithm for estimating $\text {PPV}$ on spontaneously breathing subjects. The algorithm utilizes our recently developed sequential Monte Carlo method (SMCM), which is called a maximum *a-posteriori* adaptive marginalized particle filter (MAM-PF). We report the performance assessment results of the proposed algorithm on real ABP signals from spontaneously breathing subjects.

**Results:**

Our assessment results indicate good agreement between the automatically estimated $\text {PPV}$ and the gold standard $\text {PPV}$ obtained with manual annotations. All of the automatically estimated $\text {PPV}$ index measurements ($\text {PPV}_{\text {auto}}$) were in agreement with manual gold standard measurements ($\text {PPV}_{\text {manu}}$) within ±4 % accuracy.

**Conclusion:**

The proposed automatic algorithm is able to give reliable estimations of $\text {PPV}$ given ABP signals alone during spontaneous breathing.

## Background

Excessive blood loss due to severe medical conditions can result in insufficient tissue perfusion, which can lead to organ failure. Clinicians need to plan the course of fluid therapy carefully in order to maintain tissue perfusion [1–3]. However, individuals’ responsiveness to fluid therapy varies significantly and there are few clinical signs for clinicians to rely on to predict the fluid responsiveness.

Dynamic variables such as stroke volume variation ($\text {SVV}$), systolic pressure variation ($\text {SPV}$), and pulse pressure variation ($\text {PPV}$) have been proposed as reliable indicators to guide fluid therapy in mechanically ventilated patients [4]. Particularly, $\text {PPV}$ is known as the most reliable predictor of fluid responsiveness due to its high sensitivity and specificity [5, 6]. $\text {PPV}$ attempts to quantify the degree of fluctuations in the difference between the systolic and diastolic arterial blood pressure ($\text {ABP}$). It can be calculated as follows,

1 $$\begin{aligned} \text {PPV}\,(\%) = 100 \times \frac{\mathrm {PP_{max}}-\mathrm {PP_{min}}}{(\mathrm {PP_{max}}+\mathrm {PP_{min}})/2} \end{aligned}$$

where $\mathrm {PP}_{\max }$ and $\mathrm {PP}_{\min }$ are the maximum and minimum differences between the systolic and diastolic $\text {ABP}$ over a single respiratory cycle. Several medical systems such as PICCO, Nexfin, and FloTrac are commercially available, which can compute $\text {PPV}$ under stable hemodynamic conditions [7]. The authors previously proposed a novel method that can compute $\text {PPV}$ given $\text {ABP}$ signals alone even under abruptly changing hemodynamic conditions [8]. To the best of our knowledge, however, there is no automatic algorithm for estimating $\text {PPV}$ on spontaneously breathing subjects.

Heart-lung interactions differ substantially between spontaneous breathing and mechanical ventilation. While mechanical inspiration decreases right ventricular filling and increases right ventricular afterload, spontaneous inspiration increases both right ventricular filling and afterload. Also, intrathoracic pressure oscilations during spontaneous breathing are insufficient and irregular and respiratory induced variables are not sensitive enough to evaluate the preload dependency [2, 9]. Due to this uncertainty of the usefulness of dynamic variables during spontaneous breathing, the clinical usage of dynamic variables is currently limited to predicting the fluid responsiveness of mechanically ventilated patients [10]. However, recent studies suggest that accurate prediction of the fluid responsiveness may have potential for those who are not under full mechanical ventilation support. For instance, Hong et al. [11] demonstrated that $\text {PPV}$ is of use in predicting the fluid responsiveness during forced spontaneous breathing. Forced spontaneous breathing is a special breathing exercise, which involves deep inspiration and slow passive expiration. Another study proposed the use of Dynamic Arterial Elastance (Eadyn), which is the ratio between $\text {PPV}$ and $\text {SVV}$ during a single respiratory cycle, to predict the arterial blood pressure response to a fluid challenge during post-surgical recovery periods [12]. In one porcine study, pigs breathed spontaneously into the inspiratory and expiratory threshold resistors separately or combined under three volemic conditions: hypo-, hyper-, and normo-volemic [13]. The study result indicated that expiratory resistor could be used to predict the fluid responsiveness of spontaneously breathing subjects. Hoff et al. [10] investigated the ability of respiratory variations in $\text {PPV}$ to reflect hypovolemia during noninvasive positive pressure ventilation (NPPV). They induced central hypovolemia with progressive lower body negative pressure. Their results clearly indicated that $\text {PPV}$ is associated with volume status during NPPV.

The objectives of this paper are to introduce a new algorithm for automatic estimation of $\text {PPV}$ given arterial blood pressure ($\text {ABP}$) signals alone during spontaneous breathing and to assess its performance on real $\text {ABP}$ signals from the Massachusetts General Hospital Waveform Database (MGHDB) [14] available on PhysioNet [15]. It should be noted that our previous work in [8] introduced an algorithm for automatic $\text {PPV}$ estimation for mechanically ventilated patients as opposed to the present work which is for spontaneously breathing patients.

## Methods: algorithm description

The subsequent sections explain a novel statistical signal model for ABP signals recorded from spontaneously breathing subjects and the $\text {PPV}$ index tracking algorithm utilizing our recently developed sequential Monte Carlo estimation method.

### Notation

We have adopted the notation used in [16] with minor modifications. We use boldface to denote random processes, normal face for deterministic parameters and functions, upper case letters for matrices, lower case letters for vectors and scalars, superscripts in parenthesis for particle indices, upper-case superscripts for nonlinear/linear indication, and subscripts for time indices. For example, the nonlinear portion of the state vector for the $i{\text {th}}$ state trajectory (i.e., particle) is denoted as $\varvec{x}_{n}^{\mathrm {N},(i)}$ where *n* represents the discrete time index and (*i*) denotes the $i{\text {th}}$ particle. The unnormalized particle weights are denoted as $\tilde{w}^{(i)}$ and the normalized particle weights as $w^{(i)}$. The state trajectories before resampling are denoted as $\tilde{\varvec{x}}^{(i)}_n$ and as $\varvec{x}^{(i)}_n$ after resampling.

### State-space model

The proposed automatic $\text {PPV}$ index estimator utilizes our recently developed sequential Monte Carlo estimation method which is based on the state-space model approach. The state-space model is a mathematical expression to describe the evolution of any physical system’s unobservable state $\varvec{x}_{n}$ and its relation to measurement $\varvec{y}_{n}$, where the state $\varvec{x}_{n}$ is a vector of parameters representing the system’s condition. The state-space method is a technique to estimate the state $\varvec{x}_{n}$ as a function of measurement $\varvec{y}_{n}$ utilizing the state-space model. The typical state-space model can be expressed as,

2 $$\begin{aligned} \varvec{x}_{n+1}=\,&f\left( \varvec{x}_{n}\right) +\varvec{u}_{n}\end{aligned}$$

3 $$\begin{aligned} \varvec{y}_{n} =\,&h\left( \varvec{x}_{n}\right) +\varvec{v}_{n} \end{aligned}$$

where (2) is a *process model*, (3) a *measurement model*, $f\left( \cdot \right)$ and $h\left( \cdot \right)$ functions of the state $\varvec{x}_{n}$, and $\varvec{u}_{n}$ and $\varvec{v}_{n}$ uncorrelated white noises with variances *q* and *r*. A designer needs to incorporate prior domain knowledge of a system into the state-space model and define the functions $f\left( \cdot \right)$ and $h\left( \cdot \right)$. The flexibility and versatility of the state-space method are attributable to two functions, which can be either linear or nonlinear.

#### Measurement model

The measurement model of the ABP signal is shown in (4–7), where $\varvec{\gamma }_{n}$ is the respiratory signal, $\varvec{\mu }_{n}$ the amplitude-modulated cardiac signal, $\varvec{\rho }_{k,n}$ the amplitude modulation factor of the $k{\text {th}}$ cardiac harmonic partial, $\varvec{\kappa }_{k,n}$ the $k{\text {th}}$ cardiac harmonic partial, $\varvec{\theta }^{\text {r}}_{n}$ the instantaneous respiratory angle, $\varvec{\theta }^{\text {c}}_{n}$ the instantaneous cardiac angle, $N^{\text {r}}_{\mathrm {h}}$ the number of respiratory partials, $N^{\text {c}}_{\mathrm {h}}$ the number of cardiac partials, $\varvec{v}_{n}$ the white Gaussian measurement noise with variance *r*, and $\varvec{r}_{\cdot ,k,n}, \varvec{m}_{\cdot ,k,j,n}, \varvec{c}_{\cdot ,k,n}$ the sinusoidal coefficients. This measurement model was first introduced in [17].

4 $$\begin{aligned} \varvec{y}_n=\,&\varvec{\gamma }_n+\varvec{\mu }_n+\varvec{v}_n=\varvec{\gamma }_n+\sum _{k=1}^{N^{\text {c}}_{\mathrm {h}}}\varvec{\rho }_{k,n}\varvec{\kappa }_{k,n}+\varvec{v}_n\end{aligned}$$

5 $$\begin{aligned} \varvec{\gamma }_{n}=\,&\sum _{k=1}^{N^{\text {r}}_{\mathrm {h}}}\varvec{r}_{1,k,n}\cos \left( k\varvec{\theta }^{\text {r}}_{n}\right) +\varvec{r}_{2,k,n}\sin \left( k\varvec{\theta }^{\text {r}}_{n}\right) \end{aligned}$$

6 $$\begin{aligned} \varvec{\rho }_{k,n}=\,&1+\sum _{j=1}^{N^{\text {r}}_{\mathrm {h}}}\varvec{m}_{1,k,j,n}\cos \left( j\varvec{\theta }^{\text {r}}_{n}\right) +\varvec{m}_{2,k,j,n}\sin \left( j\varvec{\theta }^{\text {r}}_{n}\right) \end{aligned}$$

7 $$\begin{aligned} \varvec{\kappa }_{k,n}=\,&\sum _{k=1}^{N^{\text {c}}_{\mathrm {h}}}\varvec{c}_{1,k,n}\cos \left( k\varvec{\theta }^{\text {c}}_n\right) +\varvec{c}_{2,k,n}\sin \left( k\varvec{\theta }^{\text {c}}_n\right) \end{aligned}$$

#### Process model

The process model describes the evolution of each element of the state $\varvec{x}_{n}$. In our application, $\varvec{x}_{n}$ includes the instantaneous respiratory and cardiac angles $\varvec{\theta }^{\text {r}}_{n}$ and $\varvec{\theta }^{\text {c}}_{n}$, the instantaneous mean respiratory $\bar{\varvec{f}}^{\text {r}}_{n}$ and cardiac $\bar{\varvec{f}}^{\text {c}}_{n}$ frequency, the instantaneous respiratory $\varvec{f}^{\text {r}}_{n}$ and cardiac $\varvec{f}^{\text {c}}_{n}$ frequencies, and the sinusoidal coefficients $\{\varvec{r}_{1,k,n},\varvec{r}_{2,k,n},\varvec{c}_{1,k,n},\varvec{c}_{2,k,n},\varvec{m}_{1,k,n},\varvec{m}_{2,k,n}\}$. The process model can be expressed as,

8 $$\begin{aligned} \varvec{\theta }^{\text {r}}_{n+1}=\,&\varvec{\theta }^{\text {r}}_{n}+2\pi T_{\mathrm {s}}\varvec{f}^{\text {r}}_{n}\end{aligned}$$

9 $$\begin{aligned} \varvec{\theta }^{\text {c}}_{n+1}=\,&\varvec{\theta }^{\text {c}}_{n}+2\pi T_{\mathrm {s}}\varvec{f}^{\text {c}}_{n}\end{aligned}$$

10 $$\begin{aligned} \bar{\varvec{f}}^{\text {r}}_{n+1}=\,&g\left[ \bar{\varvec{f}}^{\text {r}}_{n}+\varvec{u}_{\bar{\varvec{f}}^{\text {r}},n}\right] \end{aligned}$$

11 $$\begin{aligned} \bar{\varvec{f}}^{\text {c}}_{n+1}=\,&g\left[ \bar{\varvec{f}}^{\text {c}}_{n}+\varvec{u}_{\bar{\varvec{f}}^{\text {c}},n}\right] \end{aligned}$$

12 $$\begin{aligned} \varvec{f}^{\text {r}}_{n+1}=\,&\bar{\varvec{f}}^{\text {r}}_{n}+\alpha \left( \varvec{f}^{\text {r}}_{n}-\bar{\varvec{f}}^{\text {r}}_{n}\right) +\varvec{u}_{\varvec{f}^{\text {r}},n}\end{aligned}$$

13 $$\begin{aligned} \varvec{f}^{\text {c}}_{n+1}=\,&\bar{\varvec{f}}^{\text {c}}_{n}+\alpha \left( \varvec{f}^{\text {c}}_{n}-\bar{\varvec{f}}^{\text {c}}_{n}\right) +\varvec{u}_{\varvec{f}^{\text {c}},n} \end{aligned}$$

14 $$\begin{aligned} \varvec{r}_{\cdot ,k,n+1}=\,&\varvec{r}_{\cdot ,k,n}+\varvec{u}_{\varvec{r},n}\end{aligned}$$

15 $$\begin{aligned} \varvec{c}_{\cdot ,k,n+1}=\,&\varvec{c}_{\cdot ,k,n}+\varvec{u}_{\varvec{c},n}\end{aligned}$$

16 $$\begin{aligned} \varvec{m}_{\cdot ,k,n+1}=\,&\varvec{m}_{\cdot ,k,n}+\varvec{u}_{\varvec{m},n} \end{aligned}$$

where $\varvec{f}^{\text {r}}_{n}$ is the instantaneous respiratory frequency, $\varvec{f}^{\text {c}}_{n}$ the instantaneous cardiac frequency, $T_{\mathrm {s}}$ the sampling period, $\bar{\varvec{f}}^{\text {r}}_{n}$ the instantaneous mean respiratory frequency, $\bar{\varvec{f}}^{\text {c}}_{n}$ the instantaneous mean cardiac frequency, $\alpha$ the autoregressive coefficient, and $\varvec{u}_{\varvec{r},n}$, $\varvec{u}_{\varvec{c},n}$, and $\varvec{u}_{\varvec{m},n}$ the process noises with variances $q_{\varvec{r}}$, $q_{\varvec{c}}$, and $q_{\varvec{m}}$. The clipping function $g[\cdot ]$ limits the range of instantaneous mean frequencies, which can be written as,

17 $$\begin{aligned} g[f] = {\left\{ \begin{array}{ll} f_{\max } - (f-f_{\max }) &{} \quad \text {if}\; f_{\max }< f \\ f &{} \quad \text {if}\; f_{\min } < f \le f_{\max } \\ f_{\min } + (f_{\min }-f) &{} \quad \text {if}\; f \le f_{\min }. \end{array}\right. } \end{aligned}$$

The range of instantaneous mean frequencies, i.e., $\bar{\varvec{f}}^{\text {r}}_{n}$ and $\bar{\varvec{f}}^{\text {c}}_{n}$, is assumed to be known as domain knowledge.

### Maximum *A-posteriori* marginalized PF

The proposed automated $\text {PPV}$ index estimation method requires accurate estimates of the instantaneous respiratory frequency $\varvec{f}^{\text {r}}_{n}$, the instantaneous cardiac frequency $\varvec{f}^{\text {c}}_{n}$, and the morphology of an ABP signal. In order to obtain those estimates, we utilize our recently developed particle filter technique, which is called the maximum *a-posteriori* adaptive marginalized particle filter (MAM-PF). The MAM-PF is a hybrid version of the marginalized particle filter (MPF) and maximum *a-posteriori* particle filter (MAP-PF), which leverages the advantages of the MPF and MAP-PF. In [18] we described the recursions for the MAM-PF in detail. We proposed two versions of the MAM-PF: optimal and fast MAM-PFs [18]. Within the state-space method framework, the Optimal MAM-PF computes the “optimal” trajectory of the state $\varvec{x}_{n}$. However, its computational burden is too demanding to be practically useful. The fast MAM-PF is an approximation of the optimal MAM-PF, which requires dramatically less computational burden. However, the fast MAM-PF performs as well as the optimal MAM-PF, which we demonstrated in [8]. Recently, we proposed an automatic ($\text {PPV}$) estimation technique in mechanically ventilated patients by utilizing the fast MAM-PF as an ABP signal tracker [8]. Under full mechanical support, the respiratory rate of subjects is equal to the mechanical ventilation rate, which is known and constant. Therefore, the fast MAM-PF has to track only the instantaneous cardiac frequency $\varvec{f}^{\text {c}}_{n}$ along with the signal morphology.

All ABP signals included in this study were recorded from spontaneously breathing subjects. Therefore, the ABP signal tracker has to track both the instantaneous respiratory frequency $\varvec{f}^{\text {r}}_{n}$ and the instantaneous cardiac frequency $\varvec{f}^{\text {c}}_{n}$ along with the signal morphology. Although the fast MAM-PF based ABP signal tracker is capable of tracking multiple frequencies, there are two major issues in using the fast MAM-PF algorithm as the ABP signal tracker for ABP signals of spontaneously breathing subjects. The first issue is that the morphology of the signal, which is represented by the sinusoidal coefficients in (6, 7), does not belong to the linear state any more. Since the modulating signal $\varvec{\rho }_{k,n}$ is multiplied to the cardiac signal $\varvec{\kappa }_{k,n}$, their sinusoidal coefficients $\varvec{c}_{\cdot ,k,n}$ and $\varvec{m}_{\cdot ,k,j,n}$ are nonlinear parameters of the measurement model in (4). The fast MAM-PF is applicable only to state-space models whose state vector can be partitioned into the linear and nonlinear portions. The second issue is that as the dimension of the state, where particle filters are used, increases the number of necessary particles to cover the state increases exponentially. As a result, the computational burden of the fast MAM-PF increases exponentially. The portion of the state space where particle filters are used is called the *particle space*. Since the new ABP signal tracker has to estimate both the instantaneous respiratory frequency $\varvec{f}^{\text {r}}_{n}$ and the instantaneous cardiac frequency $\varvec{f}^{\text {c}}_{n}$, the dimension of the particle state becomes 2, which results in a quadruple increase of computational burden if the fast MAM-PF has to be used for the current application. In order to address these two major issues we propose a new ABP signal tracker, which is a modified version of the Fast MAM-PF. It is called, the *Dual MAM-PF*. The term “Dual” is borrowed from Dual Kalman filters, in which the state is divided into two portions and each portion is estimated separately assuming that the other portion is known and equal to the currently estimated value. While the fast MAM-PF treats a two-dimensional particle space as a whole, the dual MAM-PF partitions the two-dimensional particle space into two one-dimensional particle spaces assuming independence between two particle space variables, which are the instantaneous respiratory frequency $\varvec{f}^{\text {r}}_{n}$ and the instantaneous cardiac frequency $\varvec{f}^{\text {c}}_{n}$.

Suppose that the state vector $\varvec{x}$ can be partitioned as follows,

18 $$\begin{aligned} \varvec{x}_n = \begin{bmatrix} \varvec{x}^{\mathrm {P}}_{n} \\ \varvec{x}^{\mathrm {K}}_{n} \end{bmatrix} \end{aligned}$$

where $\varvec{x}^{\mathrm {P}}_{n}$ represents the particle state and $\varvec{x}^{\mathrm {K}}_{n}$ the Kalman state. The particle state is the portion of the state where particle filters are used as defined earlier while the Kalman state is the portion of the state where extended Kalman filters are used. The state variables whose posterior distributions are known to be multi-modal belong to the particle state while those whose posterior distributions are known to be Gaussian or uni-modal belong to the Kalman state. In [18] we demonstrated that the posterior distribution of the instantaneous frequency of a multi-harmonic signal is truly multi-modal. Given the state-space model in (4)–(7), instantaneous respiratory frequency $\varvec{f}^{\text {r}}_{n}$ and the instantaneous cardiac frequency $\varvec{f}^{\text {c}}_{n}$ are the particle state variables and the sinusoidal coefficients such as $\varvec{r}_{\cdot ,k,n}$, $\varvec{c}_{\cdot ,k,n}$, and $\varvec{m}_{\cdot ,k,j,n}$ are the Kalman state variables. Assuming that the particle state variables are independent of each other the particle state $\varvec{x}^{\mathrm {P}}_{n}$ can be partitioned further as,

19 $$\begin{aligned} \varvec{x}^{\mathrm {P}}_{n}&=\begin{bmatrix} \varvec{x}^{\mathrm {P}_{1}}_{n} \\ \varvec{x}^{\mathrm {P}_{2}}_{n} \end{bmatrix}\end{aligned}$$

20 $$\begin{aligned} \varvec{x}^{\mathrm {P}_{1}}_{n+1}&=f_{1,n}\left( \varvec{x}^{\mathrm {P}_{1}}_{n},\varvec{u}^{\mathrm {P}_{1}}_{n}\right) \end{aligned}$$

21 $$\begin{aligned} \varvec{x}^{\mathrm {P}_{2}}_{n+1}&=f_{2,n}\left( \varvec{x}^{\mathrm {P}_{2}}_{n},\varvec{u}^{\mathrm {P}_{2}}_{n}\right) \end{aligned}$$

where $\varvec{x}^{\mathrm {P}_{1}}_{n}$ and $\varvec{x}^{\mathrm {P}_{2}}_{n}$ represent the first and second particle state variables, respectively. This partitioning breaks down a two-dimensional particle space $\varvec{x}^{\mathrm {P}}_{n}$ into two one-dimensional particle spaces. The total posterior distribution is given by,

22 $$\begin{aligned} p(\varvec{x}_{0:n}|&\varvec{y}_{0:n})=p(\varvec{x}^{\mathrm {K}}_{0:n}|\varvec{y}_{0:n},\varvec{x}^{\mathrm {P}}_{0:n})p(\varvec{x}^{\mathrm {P}}_{0:n}|\varvec{y}_{0:n})\end{aligned}$$

23 $$\qquad \qquad \begin{aligned} =&\,p(\varvec{x}^{\mathrm {K}}_{0:n}|\varvec{y}_{0:n},\varvec{x}^{\mathrm {P}}_{0:n})p(\varvec{x}^{\mathrm {P}_{1}}_{0:n}|\varvec{y}_{0:n})p(\varvec{x}^{\mathrm {P}_{2}}_{0:n}|\varvec{y}_{0:n}). \end{aligned}$$

Algorithm 1 provides a complete description of the dual MAM-PF algorithm, where $N_{\mathrm {T}}$ represents the total number of signal samples, $N_{\mathrm {p}}$ the number of particles for each one-dimensional particle space, *j* the particle state variable index, and $i_{j}$ the particle index of the $j{\text {th}}$ particle state variable. The total number of particle used in the dual MAM-PF algorithm is $2 N_{\mathrm {p}}$ instead of $N_{\mathrm {p}}^{2}$. At each time step *n* the dual MAM-PF searches for the best trajectory of each particle $i_{j}$ from the previous trajectory. This searching step can be written as,

24 $$\begin{aligned} k^{*}_{j} = \underset{k_{j}}{{{\mathrm{argmax}}}}\,&\varvec{\alpha }_{j,n-1}^{(k_{j})} p \left( \varvec{y}_n|\varvec{x}_n^{\mathrm {P}_{j},(i_{j})},\hat{\varvec{x}}_{n|0:n-1}^{\mathrm {K},(k_{j})}\right) \cdots \nonumber \\&p \left( \varvec{x}_{n}^{\mathrm {P}_{j},(i_{j})}|\varvec{x}_{n-1}^{\mathrm {P}_{j},(k_{j})}\right) \end{aligned}$$

25 $$\begin{aligned} \approx \underset{k_{j}}{{{\mathrm{argmax}}}}\,&\varvec{\alpha }_{j,n-1}^{(k_{j})} p \left( \varvec{y}_n|\varvec{x}_n^{\mathrm {P}_{j},(i_{j})},\hat{\varvec{x}}_{n|0:n-1}^{\mathrm {K},(i_{j})}\right) \cdots \nonumber \\&p \left( \varvec{x}_{n}^{\mathrm {P}_{j},(i_{j})}|\varvec{x}_{n-1}^{\mathrm {P}_{j},(k_{j})}\right) \end{aligned}$$

26 $$\begin{aligned} = \underset{k_{j}}{{{\mathrm{argmax}}}}\,&\varvec{\alpha }_{j,n-1}^{(k_{j})} p \left( \varvec{x}_{n}^{\mathrm {P}_{j},(i_{j})}|\varvec{x}_{n-1}^{\mathrm {P}_{j},(k_{j})}\right) . \end{aligned}$$

Given the best trajectory for each particle $i_{j}$, corresponding Kalman state variables $\varvec{x}^{\mathrm {P}_{j},(i_{j})}$, i.e. sinusoidal coefficients, are updated utilizing the extended Kalman filter. Then, the MAP estimate of $\varvec{x}^{\mathrm {P}_{i}}_{n}$ is obtained based on the value of the coefficient $\varvec{\alpha }^{(i_j)}_{j,n}$ as follows,

27 $$\begin{aligned} i^{*}_{j,n} =\, &\underset{i_{j}}{{{\mathrm{argmax}}}}\,\varvec{\alpha }^{(i_j)}_{j,n} \end{aligned}$$

28 $$\begin{aligned} \hat{\varvec{x}}^{\mathrm {P}_{i}}_{n} =\, &\varvec{x}^{\mathrm {P}_{i},(i^{*}_{j,n})}_{n} \end{aligned}$$

Since there are two groups of particles $i_{1}$ and $i_{2}$, we need to select the best estimate of the Kalman state vector $\varvec{x}^{\mathrm {K}}_{n}$ among two potential estimates: $\varvec{x}^{\mathrm {K},(i^{*}_{1,n})}_{n}$ and $\varvec{x}^{\mathrm {K},(i^{*}_{2,n})}_{n}$. The actual estimate of the Kalman state vector $\varvec{x}^{\mathrm {K}}_{n}$ can be selected as follows,

29 $$\begin{aligned} i^{*}_{\text {MAP},n}&= {\left\{ \begin{array}{ll} i^{*}_{1,n} \quad &{} \varvec{\alpha }^{(i^{*}_{1,n})}_{1,n} \ge \varvec{\alpha }^{(i^{*}_{2,n})}_{2,n}\\ i^{*}_{2,n} \quad &{} \varvec{\alpha }^{(i^{*}_{1,n})}_{1,n} < \varvec{\alpha }^{(i^{*}_{2,n})}_{2,n}. \end{array}\right. }\end{aligned}$$

30 $$\begin{aligned} \hat{\varvec{x}}^{\mathrm {K}}_{n}&= \varvec{x}^{\mathrm {K},(i^{*}_{\text {MAP},n})}_{n} \end{aligned}$$

Then, the estimate of the entire state $\varvec{x}_{n}$ can be expressed as,

31 $$\begin{aligned} \hat{\varvec{x}}_{n}&= \{\hat{\varvec{x}}^{\mathrm {P}_{1},(i^{*}_{1,n})}_{n},\hat{\varvec{x}}^{\mathrm {P}_{2},(i^{*}_{2,n})}_{n},\hat{\varvec{x}}^{\mathrm {K},(i^{*}_{\text {MAP},n})}_{n}\} \end{aligned}$$

In order to appreciate the algorithm of the dual MAM-PF, it is essential to understand the generic particle filter along with other variants of particle filters such as the MPF, MAP-PF, and MAM-PF. We provided detail algorithms of those particle filters in [18].
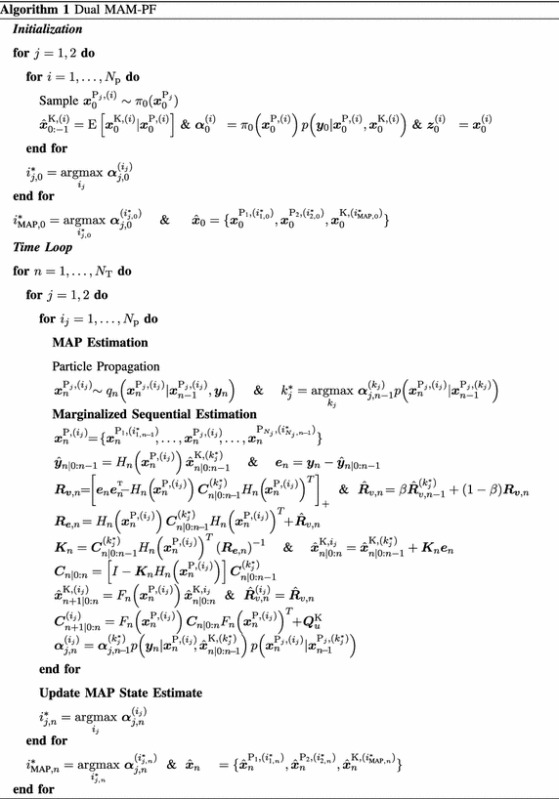


### ABP signal envelope estimation

**Fig. 1:**
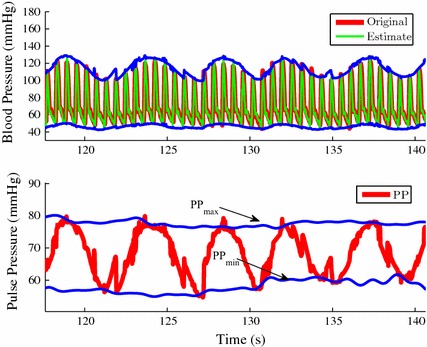
*Top* Original ABP signal (*red*) and its estimate (*green*) with automatically computed envelopes (*blue*).* Bottom* Automatically computed $\mathrm {PP}$ signal (*red*) and its envelope (*blue*)

Given the estimated signal parameters in (8–16), it is possible to estimate the upper envelope ($\varvec{e}_{\mu ,n}$) and lower envelope ($\varvec{e}_{\ell ,n}$) of ABP signals by following steps below,

32 $$\begin{aligned} \varvec{\theta }^{\text {c}}_{\max ,n}=\,&\arg \max _{\theta }\sum _{k=1}^{N^{\text {c}}_{\mathrm {h}}}\varvec{\rho }_{k,n}\left[ \varvec{c}_{1,k,n}\cos \left( k\theta \right) +\varvec{c}_{2,k,n}\sin \left( k\theta \right) \right] \nonumber \\ \varvec{\theta }^{\text {c}}_{\min ,n}=\,&\arg \min _{\theta }\sum _{k=1}^{N^{\text {c}}_{\mathrm {h}}}\varvec{\rho }_{k,n}\left[ \varvec{c}_{1,k,n}\cos \left( k\theta \right) +\varvec{c}_{2,k,n}\sin \left( k\theta \right) \right] \nonumber \\ \varvec{\kappa }_{\max ,k,n}=\,&\varvec{c}_{1,k,n}\cos \left( k\varvec{\theta }^{\text {c}}_{\max ,n}\right) +\varvec{c}_{2,k,n}\sin \left( k\varvec{\theta }^{\text {c}}_{\max ,n}\right) \nonumber \\ \varvec{\kappa }_{\min ,k,n}=\,&\varvec{c}_{1,k,n}\cos \left( k\varvec{\theta }^{\text {c}}_{\min ,n}\right) +\varvec{c}_{2,k,n}\sin \left( k\varvec{\theta }^{\text {c}}_{\min ,n}\right) \nonumber \\ \varvec{e}_{\mu ,n}=\,&\varvec{\gamma }_{n}\,+\,\sum _{k=1}^{N^{\text {c}}_{\mathrm {h}}}\varvec{\rho }_{k,n}\varvec{\kappa }_{\max ,k,n}\end{aligned}$$

33 $$\begin{aligned} \varvec{e}_{\ell ,n}=\,&\varvec{\gamma }_{n}\,+\,\sum _{k=1}^{N^{\text {c}}_{\mathrm {h}}}\varvec{\rho }_{k,n}\varvec{\kappa }_{\min ,k,n} \end{aligned}$$

where $\arg \max _{x} f(x)$ and $\arg \min _{x} f(x)$ are operators to obtain the value of *x* for which *f*(*x*) attains its maximum and minimum values, respectively. The top plot in Fig. 1 shows a five respiratory cycle period of an ABP signal $\varvec{y}_{n}$ (thick red), its estimate $\hat{\varvec{y}}_{n}$ (thin green), and its estimated envelopes $\varvec{e}_{\mu ,n}$ and $\varvec{e}_{\ell ,n}$ (blue), which are described in (32) and (33).

### Pulse pressure signal envelope estimation

The pulse pressure ($\mathrm {PP}$) signal is the difference between the upper envelope $\varvec{e}_{\mu ,n}$ and lower envelope $\varvec{e}_{\ell ,n}$ of the ABP signal. This $\mathrm {PP}$ signal oscillates roughly at the respiratory rate as shown in the bottom plot in Fig. 1. This oscillation is due to the respiratory effect on the variation of systemic ABP under full ventilation support [19]. Within each respiratory cycle $\mathrm {PP}$ reaches its maximum ($\mathrm {PP}_{\max }$) and minimum ($\mathrm {PP}_{\min }$) values, which are two critical parameters to compute the $\text {PPV}$ index. Traditionally, the $\mathrm {PP}_{\max }$ and $\mathrm {PP}_{\min }$ values have been computed only once per each respiratory cycle. Given the estimated signal parameters in (8–16), however, we can compute the continuous equivalents of $\mathrm {PP}_{\max }$ and $\mathrm {PP}_{\min }$. They are the upper $\varvec{\varepsilon }_{\mu ,n}$ and lower $\varvec{\varepsilon }_{\ell ,n}$ envelopes of the $\mathrm {PP}$ signal. The upper envelope $\varvec{\varepsilon }_{\mu ,n}$ is the continuous estimate of $\mathrm {PP}_{\max }$ and the lower envelope $\varvec{\varepsilon }_{\ell ,n}$ that of $\mathrm {PP}_{\min }$. The $\varvec{\varepsilon }_{\mu ,n}$ and $\varvec{\varepsilon }_{\ell ,n}$ values can be estimated as described below,

34 $$\begin{aligned} \varvec{\varrho }_{k,n}&=\sum _{j=1}^{N^{\text {r}}_{\mathrm {h}}}\varvec{m}_{1,k,j,n}\cos \left( j\theta \right) +\varvec{m}_{2,k,j,n}\sin \left( j\theta \right) \nonumber \\ \varvec{\theta }^{\text {r}}_{\max ,n}&=\arg \max _{\theta }\sum _{k=1}^{N^{\text {c}}_{\mathrm {h}}}\left( 1+\varvec{\varrho }_{k,n}\right) \left( \varvec{\kappa }_{\max ,k,n}  -  \varvec{\kappa }_{\min ,k,n}\right) \nonumber \\ \varvec{\theta }^{\text {r}}_{\min ,n}&=\arg \min _{\theta }\sum _{k=1}^{N^{\text {c}}_{\mathrm {h}}}\left( 1+\varvec{\varrho }_{k,n}\right) \left( \varvec{\kappa }_{\max ,k,n}  -  \varvec{\kappa }_{\min ,k,n}\right) \nonumber \\ \varvec{\varrho }_{\max ,k,n}&= \sum _{j=1}^{N^{\text {r}}_{\mathrm {h}}}\varvec{m}_{1,k,j,n}\cos \left( j\varvec{\theta }^{\text {r}}_{\max ,n}\right)   +  \varvec{m}_{2,k,j,n}\sin \left( j\varvec{\theta }^{\text {r}}_{\max ,n}\right) \nonumber \\ \varvec{\varrho }_{\min ,k,n}  &=  \sum _{j=1}^{N^{\text {r}}_{\mathrm {h}}}\varvec{m}_{1,k,j,n}\cos \left( j\varvec{\theta }^{\text {r}}_{\min ,n}\right)   +  \varvec{m}_{2,k,j,n}\sin \left( j\varvec{\theta }^{\text {r}}_{\min ,n}\right) \nonumber \\ \varvec{\varepsilon }_{\mu ,n}&=\sum _{k=1}^{N^{\text {c}}_{\mathrm {h}}}\left( 1+\varvec{\varrho }_{\max ,k,n}\right) \left( \varvec{\kappa }_{\max ,k,n}  -  \varvec{\kappa }_{\min ,k,n}\right) \end{aligned}$$

35 $$\begin{aligned} \varvec{\varepsilon }_{\ell ,n}&=\sum _{k=1}^{N^{\text {c}}_{\mathrm {h}}}\left( 1+\varvec{\varrho }_{\min ,k,n}\right) \left( \varvec{\kappa }_{\max ,k,n}  -  \varvec{\kappa }_{\min ,k,n}\right) \end{aligned}$$

where $1+\varvec{\varrho }_{k,n}$ is equal to $\varvec{\rho }_{k,n}$ and $\varvec{\varepsilon }_{\mu ,n}$ and $\varvec{\varepsilon }_{\ell ,n}$ are the continuous estimates of the $\mathrm {PP}_{\max }$ and $\mathrm {PP}_{\min }$, respectively. The blue lines in the bottom plot in Fig. 1 represent the upper $\varvec{\varepsilon }_{\mu ,n}$ and lower $\varvec{\varepsilon }_{\ell ,n}$ envelopes of the $\mathrm {PP}$ signal, which are obtained by following the method described above.

**Fig. 2:**
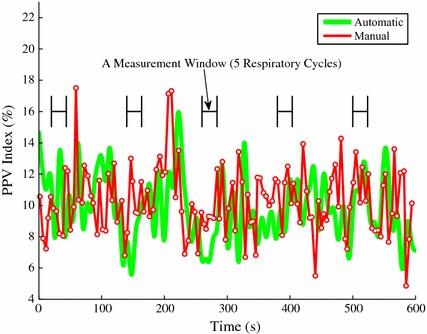
Automatic $\text {PPV}$ index (*green*) and manual $\text {PPV}$ index (*red*) over the entire ABP signal duration (10 min). One $\text {PPV}$ index measurement is computed from each measurement window, which is a time period of five respiratory cycles

### Pulse pressure variation calculation

Given the $\varvec{\varepsilon }_{\mu ,n}$ and lower $\varvec{\varepsilon }_{\ell ,n}$ values, it is straightforward to calculate the $\text {PPV}$ index. It can be computed as follows,

36 $$\begin{aligned} \text {PPV}\,(\%)=\,&100\times \frac{\varvec{\varepsilon }_{\max }-\varvec{\varepsilon }_{min}}{(\varvec{\varepsilon }_{\max }+\varvec{\varepsilon }_{\min })/2} \end{aligned}$$

This new $\text {PPV}$ index is different from the traditional $\text {PPV}$ index described in (1) because the new one is continuous in time while the traditional one can be obtained only once per each respiratory cycle.

Figure 2 illustrates an example of the automatically computed continuous $\text {PPV}$ index (thick green) and the manually obtained discrete $\text {PPV}$ index (thin red) of a real 10 min ABP signal. Each hollow white dot represents a “discrete” $\text {PPV}$ index, which can be obtained once per each respiratory cycle.

The subsequent sections describe how to assess the accuracy of the proposed $\text {PPV}$ index tracking algorithm.

## Methods: algorithm assessment

### Assessment data

**Fig. 3:**
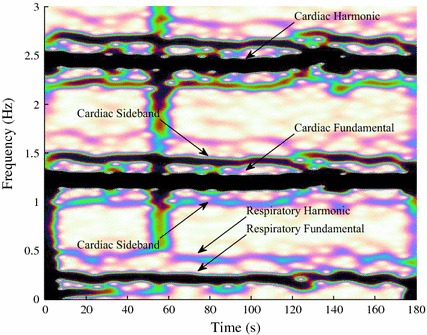
Spectrogram of one of the 11 ABP signals recorded from spontaneously breathing subjects

The Massachusetts General Hospital waveform database (MGHDB) on PhysioNet is a comprehensive collection of electronic recordings of hemodynamic and electrocardiographic waveforms patients in critical care units [14, 15]. It consists of recordings from 250 patients representing a broad spectrum of physiologic and pathophysiologic states. The typical recording includes arterial blood pressure (ABP) signals in addition to seven other types of waveforms. By visually inspecting the spectrogram and time-series of ABP signals we identified 11 patients who breathed spontaneously. The first column in Table 2 lists the patients’ identification numbers (e.g. mgh000) as in MGHDB on Physionet. Figure 3 shows the spectrogram of one of the 11 ABP signals. Each ABP signal is 10 min long and the total duration of the 11 ABP signals was 2 h. The original sample rate $f_{\text {s}}$ of the signals was 360 Hz, but they were downsampled by a factor of 9, so that the final sample rate $f_{\text {s}}$ was 40 Hz.

The number of cardiac components $N^{\text {c}}$ was 5 and that of respiratory components $N^{\text {r}}$ was 2. The total number of particles $2 N^{\mathrm {p}}$ was 500. Table 1 lists the parameter values used for the $\text {PPV}$ index estimator. Those parameter values were initialized and tuned based on the previously published work, where ABP signals were recorded during full mechanical ventilation [8].

**Table 1:** Summary of user-specified design parameters for the $\text {PPV}$ index tracker

Name	Symbol	Value
No. particles	$2 N^{\mathrm {p}}$	500
No. cardiac components	$N^{\text {c}}$	10
No. respiratory components	$N^{\text {r}}$	3
Minimum respiratory rate	$f^{\text {r}}_{\min }$	6/60 Hz
Maximum respiratory rate	$f^{\text {r}}_{\max }$	30/60 Hz
Minimum heart rate	$f^{\text {c}}_{\min }$	50/60 Hz
Maximum heart rate	$f^{\text {c}}_{\max }$	140/60 Hz
Measurement noise variance	*r*	${{\mathrm{var}}}(y)$/1e3
Respiratory frequency variance	$q_{f^{\text {r}}}$	1e−6 $T_s$
Cardiac frequency variance	$q_{f^{\text {c}}}$	1e−6 $T_s$
Respiratory amplitude variance	$q_{a}, q_{b}$	${{\mathrm{var}}}(y)$1e−6$T_s$
Modulation factor amplitude variance	$q_{c}, q_{d}$	${{\mathrm{var}}}(y)$1e−8$T_s$
Cardiac amplitude variance	$q_{e}, q_{f}$	${{\mathrm{var}}}(y)$1e−6$T_s$
Initial respiratory amplitude	$u_{a}, u_{b}$	${{\mathrm{std}}}(y)/1e1$
Initial modulation factor amplitude	$u_{c}, u_{d}$	${{\mathrm{std}}}(y)/1e3$
Initial cardiac amplitude	$u_{e}, u_{f}$	${{\mathrm{std}}}(y)/1e1$

### Manual PPV annotations (current standard)

We manually annotated the peaks and troughs of the ABP signals and calculated the $\text {PPV}$ indices (current standard) as defined in (1). They are referred to as manual $\text {PPV}$ indices $\text {PPV}_{\text {manu}}$. $\text {PPV}_{\text {auto}}$ represents $\text {PPV}$ indices obtained using the proposed $\text {PPV}$ index tracking algorithm.

### Statistical analysis

The statistical analysis used five $\text {PPV}$ index measurements for each subject, and each measurement was separated by 2 min. Each $\text {PPV}$ index measurement is an averaged value over 5 respiratory cycles. Figure 2 shows the 2 min apart measurement periods. The proposed $\text {PPV}$ index tracking algorithm was assessed by calculating the agreement (mean ± standard deviation) between $\text {PPV}_{\text {auto}}$ and $\text {PPV}_{\text {manu}}$ measurements and using Bland–Altman analysis.

A Bland-Altman plot is a statistical and visualization method that is often used in the assessment of $\text {PPV}$ estimation algorithms in order to determine the agreement between two different $\text {PPV}$ estimates. It has the difference $\Delta \text {PPV}$ between $\text {PPV}_{\text {auto}}$ and $\text {PPV}_{\text {manu}}$ on the y-axis and the $\text {PPV}_{\text {manu}}$ on the x-axis. It visualizes the overall accuracy of estimation and estimation bias or trend versus $\text {PPV}_{\text {manu}}$. We used it to compare the current standard using manual annotations with our automatic estimation algorithm.

## Results

**Fig. 4:**
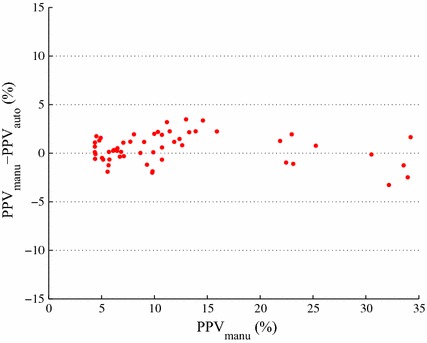
Bland–Altman plot of the 11 subjects

Figure 4 depicts the Bland–Altman plot of the 11 subjects. There are 5 $\text {PPV}$ measurements available per each subject. All of $\text {PPV}_{\text {auto}}$ measurements were in agreement with $\text {PPV}_{\text {manu}}$ measurements within ±3.5 % accuracy.

Table 2 lists the mean ± standard deviation of 5 $\text {PPV}_{\text {manu}}$ and $\text {PPV}_{\text {auto}}$ measurements for each subject. The second column is for $\text {PPV}_{\text {manu}}$ and the third column for $\text {PPV}_{\text {auto}}$.

**Table 2:** Summary of the mean and standard deviation of the $\text {PPV}_{\text {manu}}$ and $\text {PPV}_{\text {auto}}$ measurements

Subject	$\text {PPV}_{\text {manu}}$ (%)	$\text {PPV}_{\text {auto}}$ (%)
1 (mgh003)	9.8 ± 1.0	8.9 ± 1.0
2 (mgh007)	32.9 ± 1.5	33.9 ± 2.3
3 (mgh011)	10.3 ± 1.0	10.6 ± 0.8
4 (mgh091)	4.6 ± 0.3	3.3 ± 0.3
5 (mgh092)	10.3 ± 1.1	9.2 ± 1.3
6 (mgh151)	12.9 ± 2.7	11.3 ± 2.0
7 (mgh152)	5.9 ± 0.5	6.1 ± 0.5
8 (mgh158)	12.5 ± 0.8	10.3 ± 1.5
9 (mgh164)	7.6 ± 1.0	6.7 ± 0.9
10 (mgh169)	4.8 ± 0.5	5.3 ± 1.3
11 (mgh183)	6.5 ± 0.8	6.5 ± 0.6

## Discussion

### Frequency clipping function

The clipping function $g[\cdot ]$ in (17) could be defined as follows,

37 $$\begin{aligned} g[f] = {\left\{ \begin{array}{ll} f_{\max } &{} \quad \text {if}\; f_{\max }< f \\ f  &{} \quad \text {if}\; f_{\min } < f \le f_{\max } \\ f_{\min } &{} \quad \text {if}\; f \le f_{\min }. \end{array}\right. } \end{aligned}$$

However, there is a major problem with the clipping function in (37) when it is incorporated into the particle filter framework. It tends to cause the boundary values, $f_{\max }$ and $f_{\min }$, to be the pitfalls for the particles, $\varvec{x}^{\mathrm {P}_{1}}_{n}$ and $\varvec{x}^{\mathrm {P}_{2}}_{n}$, to be trapped in when the instantaneous frequency values associated with the particles become close to $f_{\max }$ or $f_{\min }$. In other words, once any particle’s frequency value meets either of the boundary conditions, the particle tends to remain in the same state having the frequency value of either $f_{\max }$ or $f_{\min }$. In order to address this issue, the clipping function is defined as shown in (17), which forces the frequency value to bounce back within the range, i.e., $f_{\min } < f \le f_{\max }$, once it reaches beyond $f_{\max }$ and $f_{\min }$.

### Algorithm’s advantages

The proposed algorithm is the first automatic method described in the literature especially designed to estimate and track the $\text {PPV}$ index in situations involving spontaneous breathing. It is important to note that the proposed algorithm is a complete new design from our previously described algorithm [20] which only worked for mechanically ventilated subjects. Our previous algorithm was made publicly available by the authors and due to its performance has been adopted by Philips Medical Systems. Currently, our previously published PPV algorithm is displayed in real-time on the Philips Intelliveu MP70 monitors (Intellivue MP70, Philips Medical Systems) and has been used in numerous studies related to PPV and fluid responsiveness. Its ability to monitor fluid responsiveness in the operating room and its accuracy against the current standard obtained by manual annotations were assessed by Cannesson [21]. Previously it was not possible to conveniently monitor the $\text {PPV}$ index in the operating room or in the intensive care unit because it had to be manually calculated. Thus, the automatic PPV has potential clinical application for fluid management optimization in the operating room.

A limitation of our previously described algorithm adopted by Philips in their Intelliveu MP70 monitors is that it may not work adequately in regions of abrupt hemodynamic changes [20] and it is only accurate for mechanically ventilated subjects. In this paper, we provide a detailed description of a novel algorithm designed to be a robust PPV estimator during regions of abrupt hemodynamic changes and during spontaneous breathing.

**Fig. 5:**
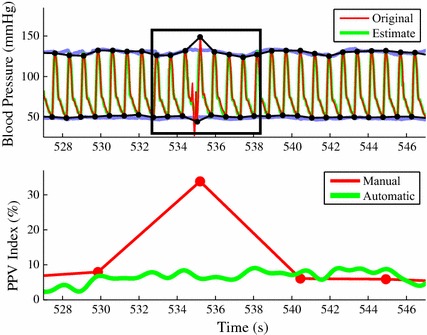
*Top* Original ABP signal (*red*) with its manually annotated envelopes (*black*) and signal estimates (*green*) with its automatically computed envelopes (*purple*).* Bottom* Manual $\text {PPV}$ indices (*red*) and automatic $\text {PPV}$ indices (*green*) where one of the manual $\text {PPV}$ indices has an abnormally high value

**Fig. 6:**
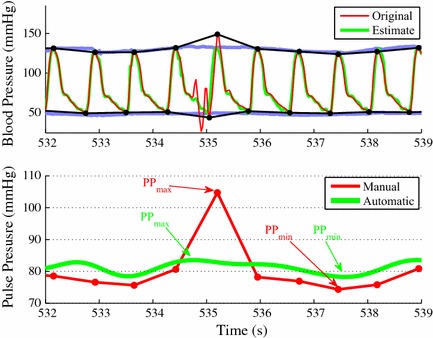
*Top* Original ABP signal (*red*) with its manually annotated envelopes (*black*) and signal estimates (*green*) with its automatically computed envelopes (*purple*).* Bottom* Manual $\mathrm {PP}$ signal (*red*) and automatic $\mathrm {PP}$ signal (*green*) where the manual $\mathrm {PP}$ signal increases momentarily due to a contaminated heart beat in the ABP signal

The major algorithm design difference of the proposed algorithm with respect to previously published algorithms [20, 22] is the fact that the proposed method is based on a statistical state-space capable of modeling spontaneous breathing, and estimation of the cardiovascular pressure signal based on this statistical model using optimal estimation methods. The state-space modeling stage results in an algorithm that is more robust to hemodynamic changes and artifacts. The statistical state-space signal model and associated model parameter estimation algorithm automatically filter out noise and artifact that cannot be captured with the model. Since the statistical signal model is based on cardiovascular physiology and pathophysiology, signal features that are not physiological in nature are automatically filtered out. Additionally, the model is general enough to accurately model both arterial blood pressure signals and plethysmogram signals. Consequently, it can also be used to calculate the pleth variability index (PVI).

Figures 5 and 6 exemplify a case where signal features that are not physiological in nature are automatically filtered out resulting in more accurate $\text {PPV}$ index estimation than manual annotation. The top plot in Fig. 5 illustrates 4 respiratory cycles of the ABP signal (red) and its estimate (green). It also shows the manually annotated signal envelopes (black) and the automated computed signal envelopes (light blue). The bottom plot in Fig. 5 depicts the $\text {PPV}_{\text {manu}}$ and $\text {PPV}_{\text {auto}}$ over the same period. Around $535\,\mathrm{s}$, the $\text {PPV}_{\text {manu}}$ value (red) abruptly increases up to $35\%$ while the $\text {PPV}_{\text {auto}}$ value (green) remains at 8 %. Around 540 s, the $\text {PPV}_{\text {manu}}$ value returns to 8 %. Figure 6 focuses on the time period marked with the black rectangular box in Fig. 5. The top plot in Fig. 6 shows that the heart beat between 535 and 535.5 s is contaminated by noise and has an abnormal morphology. As a result, the corresponding $\mathrm {PP}_{\text {manu}}$ shown in the bottom plot reaches a large maximum value ($\mathrm {PP}_{\text {min,manu}}:105 \mathrm{\, mm\, Hg}$) around at 535 s. However, the automatically computed maximum $\mathrm {PP}$ value ($\mathrm {PP}_{\text {min, auto}}$) at the same time is as low as $83 \mathrm{\, mm\, Hg}$. This discrepancy between the manual annotation and the proposed automatic method results from the capability of the MAM-PF algorithm, which estimates the ABP signal based on the state-space model. While the original heart beat between 535 and 535.5 s in Fig. 6 is abnormal in a physiological sense, the estimated heart beat over the same time period shows the physiologically expected morphology and location of the heart beat.

### Study limitations

The algorithm’s assessment was based on only 11 subjects with pre-recorded ABP data. Additionally, for each subject five $\text {PPV}$ estimates were used in the assessment study. This assessment was designed to be an engineering algorithm validation against current standard manual annotations, and not a clinical validation study. Consequently, a clinical validation study assessing the ability of the proposed algorithm to monitor fluid responsiveness in the operating room in situations involving spontaneously breathing subjects still needs to be conducted. This may require the proposed algorithm to be first adopted as part of a commercial system as was the case with our previous automatic $\text {PPV}$ algorithm [20].

## Conclusion

We have described the first automatic $\text {PPV}$ tracking algorithm for spontaneously breathing subjects. This novel algorithms is based on a statistical state-space model inspired in the underlying cardiovascular and respiratory physiology. This algorithm uses our recently developed SMCM (MAM-PF) for optimal parameter estimation. The assessment results indicate good agreement against the current standard $\text {PPV}$. The algorithm was designed to work during regions of abrupt hemodynamic changes and spontaneous breathing. All of $\text {PPV}_{\text {auto}}$ measurements were in agreement with $\text {PPV}_{\text {manu}}$ measurements within ±4 % accuracy.
